# The impact of older employees’ generativity on job dedication: a socioemotional selectivity perspective

**DOI:** 10.3389/fpsyg.2026.1703410

**Published:** 2026-01-20

**Authors:** Zhenxing Gong, Yongqi He, Miaomiao Li

**Affiliations:** 1 School of Business, Liaocheng University, Liaocheng, China; 2 Business School, Beijing Information Science and Technology University, Beijing, China

**Keywords:** generativity, job dedication, organizational commitment, socioemotional selectivity theory, trust

## Abstract

**Introduction:**

As organizations worldwide grapple with an aging workforce and extended careers, understanding and enhancing the psychological mechanisms underpinning older employees’ job dedication has become increasingly crucial. Building upon Carstensen’s socioemotional selectivity theory, this study explores how older employees’ generativity translates into job dedication through organizational commitment, with trust serving as a key boundary condition.

**Methods:**

We address this gap through a three-wave longitudinal study of 489 older employees across multiple organizations in eastern China. Using exploratory factor analysis, confirmatory factor analysis, correlation analysis, and Bootstrap mediation testing, we examine the proposed relationships.

**Results:**

The findings indicate that: (1) generativity exerts a significant positive effect on job dedication (*β* = 1.06, *p* < 0.01); (2) generativity promotes job dedication by influencing organizational commitment (*β* = 0.99, *p* < 0.01), which in turn enhances job dedication (*β* = 0.61, *p* < 0.01); (3) cognitive trust (*β* = 0.15, p < 0.01) and affective trust (*β* = 0.18, *p* < 0.01) significantly moderate the effect of generativity on organizational commitment.

**Discussion:**

Theoretically, our findings extend the socioemotional selectivity theory by demonstrating how generativity acts as a psychological bridge between older employees’ future time perspective and their job dedication. Practically, we identified specific trust-building practices that organizations can implement to unlock the generativity motivation potential of older employees. Thus, this study provides theoretical advances and actionable insights for developing age-inclusive workplaces.

## 1 Introduction

As China’s statutory retirement age gradually increases, critical sectors, such as hospitals, universities, and manufacturing, face the reality of a large number of older employees remaining in the workforce. However, phenomena like “idle expertise,” “generational disconnect,” and “marginalization of innovation” are prevalent in practice. Many older employees possess extensive knowledge but gradually become alienated from their organizations due to a lack of trusted creative space (Guo et al., 2023). This not only diminishes their job dedication but also leads to the loss of organizational tacit knowledge and a talent gap. More critically, when older employees’ efforts go unrecognized or unsupported, it may trigger generativity frustration. This not only reduces their retention intent but can also spread negative emotions, damaging team morale (Serrat et al., 2021). Against this backdrop, transforming older employees’ intrinsic developmental needs into sustainable organizational contributions has become an urgent management challenge. Individuals have a fundamental psychological need to develop and maintain a personal profile that can be perpetuated and immortalized (Sedikides, 2021). Generativity originates from Erikson’s psychosocial development theory, referring to an individual’s intrinsic motivation and behavioral tendency to guide and nurture the next generation, creating value for posterity (McAdams and de St Aubin, 1992). Within organizational settings, generativity manifests as older employees fulfilling their psychological need for self-transcendence through knowledge transfer, experience sharing, and mentoring younger colleagues. This concept fundamentally differs from conventional notions of “creativity”: generativity emphasizes altruism and legacy, focusing on long-term positive impacts on others and society rather than short-term problem-solving or innovative outputs. As individuals age, their generativity needs significantly intensify, becoming one of the core motivations driving older employees’ work behaviors (Wiktorowicz et al., 2022). Older employees share their knowledge and skills with younger employees (Wang et al., 2023), and both individual and organizational generativity are enhanced, helping to create prospects for future career success. Just as Erikson’s theory of psychosocial development locates generativity as the seventh of the eight developmental tasks in life, it is hypothesized that it is particularly prominent in midlife (i.e., between the ages of approximately 40 and 65) (Erikson, 1963). McAdams and McLean (2013) introduced the study of generativity into the field of organizational management and found that the generativity of older employees improved interpersonal relationships, increased dedication and dynamism, and motivated people to take action and share their knowledge and experience with others. Generativity was found to be positively associated with job satisfaction, job dedication, and leadership effectiveness among older employees (Clark and Arnold, 2008; Kooij et al., 2013; Zacher et al., 2011).

Although the generativity of older employees provides a new entry point for the adequate development and utilization of human resources in the context of an ageing workplace, and in particular for the improvement of the job dedication of older employees, however, the positive impact of the generativity of older employees on the job dedication has not been taken for granted as much as it intuitively feels. On the one hand, it has been found that opportunities for older employees to demonstrate generativity can satisfy the individual needs of older employees (Peisah et al., 2009), increase job satisfaction and job dedication (Peng et al., 2020), demonstrate equality of intergenerational contact (Yaghoobzadeh et al., 2020), and reduce prejudice (Bradley, 1997). On the other hand, studies have proposed that generativity can compromise the public interest by satisfying individual interests and reducing job dedication (Doerwald et al., 2021), and that generativity brings intergenerational conflict and negative experiences that increase costs for older employees (Maulod et al., 2023). These conflicting views and empirical results raise an unresolved dilemma as to whether employers should improve the job dedication of older employees by increasing their generativity. However, existing research lacks explanations for these inconsistent findings, leading to imperfect research on the consequences of generativity and failing to provide effective guidance for improving the job dedication of older employees. The reason for this is that although the above studies have tried to argue the influence mechanism of generativity from different theoretical perspectives, they are still fundamentally based on the social exchange theory, the underlying assumption of which is that employees who pay will be rewarded, and those who are rewarded will double their efforts, and vice versa; those who pay with no reward but increase their costs will pay less (Cropanzano and Mitchell, 2005). Despite the widespread influence of social exchange theory in explaining workplace behavior (Love and Forret, 2008), recent studies have confirmed its theoretical limitations when applied to older employees. First, social exchange theory posits that behavior is primarily driven by explicit cost–benefit calculations. However, research indicates a weak association between older employees’ knowledge-sharing behaviors and their anticipated returns—a finding that significantly diverges from the theory’s core assumptions (Tang and Martins, 2021). Second, social exchange theory struggles to explain why older employees persist in generativity even in environments with low organizational support. Many older employees continue knowledge transfer activities primarily to satisfy intrinsic emotional needs rather than external rewards (Muzafary et al., 2021). Third, while social exchange theory emphasizes reciprocity and long-term resource accumulation, individuals’ goal structures often shift from future-oriented to emotion-satisfaction-oriented with advancing age (Wiktorowicz et al., 2022). This shift makes the socioemotional selectivity theory (SST) a more fitting theoretical framework for older employees’ psychological and behavioral characteristics. SST posits that when individuals perceive limited future time, they prioritize emotional meaning and present satisfaction over long-term resource maximization (Carstensen, 2021). This perspective aligns strongly with the intrinsic motivation of generativity among older employees, offering a more appropriate theoretical lens for understanding generativity in this demographic (Wiktorowicz et al., 2022).

The socioemotional selectivity theory (SST) suggests that an individual’s perception of remaining time affects the goals people pursue, and that as people grow older and their time horizons are limited, their goals are gradually tilted toward the emotional, and they focus more on easy-to-achieve, low-risk short-term goals (Cropanzano and Mitchell, 2005). “Warm glow” emphasizes that altruistic behaviors that contribute resources for the benefit of others can contribute to individual satisfaction and identification with the organization, that people who care about others are happier than those who only care about their interests, and that the resulting positive emotional experience increases the occurrence of altruistic behaviors (Isen, 1970). Older employees place more emphasis on emotional satisfaction, especially identification with the unit of employment and its values (Peisah et al., 2009), will devote more resources to emotionally meaningful goals and activities (Löckenhoff and Carstensen, 2004), social interactions are selectively narrowed to maximize positive emotions, and social networks are systematically improved (Lubben, 1988). At the same time, the study concluded that the effect of generativity is influenced by cultural factors, with individualistic countries focusing on independence and emotional self-evaluation, while collectivism pays more attention to relationships and inter-individual collaboration; however, previous studies focusing on Western countries have lacked attention to collectivist cultures, resulting in a limited understanding of the cultural contextual factors that influence the effect of generativity.

Given the inconsistent findings regarding the relationship between generativity and job dedication, insufficient attention to the unique characteristics of older employees, and limited understanding of the boundary conditions for this relationship, this study aims to systematically address these theoretical discrepancies and practical uncertainties. Grounded in the socioemotional selectivity theory, it seeks to explicitly answer the following three core questions: (1) What is the direct effect of generativity on job dedication? (2) Does generativity positively influence older employees’ job dedication through organizational commitment? (3) Do cognitive trust and affective trust moderate generativity’s effect on organizational commitment, thereby altering the strength of the mediating path? The theoretical contributions of this study are threefold: First, it transcends the limitations of traditional social exchange theory by offering a new theoretical framework—grounded in age-specific needs—to explain the inconsistent findings between generativity and job dedication. Second, it reveals the underlying psychological mechanisms through which generativity influences older employees’ job dedication, enriching our understanding of their motivational structure. Third, it explores the differential roles of cognitive trust and affective trust within a collectivist cultural context, expanding the cultural boundaries of generativity research. Practically, this study offers targeted management insights for organizations navigating demographic aging challenges, assisting them in designing human resource strategies better aligned with older employees’ needs. This approach maximizes the value of older talent and fosters sustainable organizational development.

## 2 Literature review and hypothesis formulation

### 2.1 Older employees’ generativity and job dedication

Generativity refers to experiences that energize and invigorate individuals, potentially yielding lasting impacts and transformative outcomes (Thomas and Tee, 2022). Within organizations, older employees leverage generativity to mentor and guide younger colleagues, transmit valuable standards and values, enhance relationship quality among team members, and infuse positive energy and vitality (Gong and Li, 2024). Fasbender and Gerpott’s (2022) research indicates that Work provides older employees opportunities to share work provides older employees opportunities to share knowledge and experience with younger colleagues while transmitting ideas (Fasbender and Gerpott, 2022). Based on the socioemotional selectivity theory, generativity is particularly crucial for older employees’ job dedication (Wiktorowicz et al., 2022). Job dedication represents an employee’s psychological state of wholehearted commitment to their role, integrating their positive self with their work identity. It encompasses work vitality, dedication, and focus.

First, social-emotional selectivity theory posits that positive emotions stem from meaningful (age-appropriate) behaviors, and generativity enhances individuals’ perception of work meaning, thereby boosting work vitality (Charles, 2022). Intrinsic goals drive generativity, which in turn fosters engagement in workplace tasks to sustain work vitality (Fasbender et al., 2021). Transferring skills and knowledge enhances perceived job control, a key source of work engagement (Salamon et al., 2021).

Second, pursuing generativity—the fulfillment of intrinsic goals—meets the fundamental psychological needs of older employees (Wang et al., 2024), facilitating their continued contributions to the organization. Generativity in work involves the focus and actions of leaving a lasting legacy (Wiktorowicz et al., 2022). It may positively influence employees’ perceptions of future time. When older employees perceive sufficient time and opportunities remaining in their careers, they tend to view future career development more optimistically (Jia et al., 2024), thereby enhancing their career satisfaction.

Third, social-emotional selectivity theory posits that as people age, they pursue goals more selectively. Older employees prioritize goals that bring meaning and positive emotions, while younger individuals are more knowledge-acquisition oriented. When time is perceived as finite, emotionally fulfilling and meaningful goals become more relevant. Mentorship serves as one vehicle for generativity. Research indicates that older employees are motivated to invest resources in younger employees and develop high-quality relationships (Fasbender and Gerpott, 2022). Qualitative research by Allen et al. (1997) found that mentors guiding mentees helps build supportive networks within organizations, while mentees provide mentors with channels to express thoughts and feelings. This fulfills mentors’ desire for generativity outcomes and enhances their work focus (Quew-Jones and Rowe, 2022). Generativity can provide resources to prevent role strain (Wiktorowicz et al., 2022), helping older employees adapt to short-term goal fulfillment amid age-related changes and enhancing their work focus. Based on the above discussion, the following hypothesis is proposed:

*H1*: Generativity positively influences older employees' job dedication.

### 2.2 The mediating role of organizational commitment

Social-emotional selectivity theory centers on the future time perspective (Charles, 2022), indicating that as older employees age, their perception of future time shortens. Their work priorities shift from achieving developmental goals to pursuing social-emotional or generative goals (Zhang and Wood, 2025), elevating the importance of short-term attainable objectives. Older employees increasingly value close social relationships and yearn to “leave a mark.” Generativity in the workplace positively contributes to employee wellbeing and organizational outcomes (Wiktorowicz et al., 2022). Organizational commitment refers to an individual’s identification with an organization’s goals and values, along with associated positive emotional experiences. It comprises affective commitment, continuance commitment, and normative commitment (Muthuveloo and Rose, 2005). Affective commitment represents an attitudinal component, while continuance and normative commitments fall under behavioral domains, predicting employee turnover behavior.

Generativity positively influences organizational commitment through the following mechanisms:

First, affective commitment involves identification with and acceptance of organizational goals and values. Generativity provides individuals with identity and work meaning, thereby positively influencing affective commitment. Generativity motivates older employees to invest time and effort in relationships with younger employees (Fasbender et al., 2021) and correlates positively with post-retirement job satisfaction and perceived organizational contribution (Pundt et al., 2015), fulfilling the need to contribute to work. Ding and Schuett (2020) found that higher generativity correlates with stronger perceived work meaning, greater job satisfaction, and increased affective commitment among older employees (Ding and Schuett, 2020).

Second, sustained commitment arises from recognizing sunk costs due to increased organizational investment, leading to a willingness to remain with the organization (Xia et al., 2022). Klooker and Hölzle (2024) posit that generativity fosters organizational commitment by creating, preserving, and delivering value (Klooker and Hölzle, 2024). Although older employees may exhibit lower digital work performance than their younger counterparts, potentially affecting engagement, their accumulated human capital enables them to execute tasks proficiently, acquire new skills, and maintain productivity. Older employees demonstrate sustained commitment to fostering younger colleagues’ development by serving as mentors or advisors while exerting influence, embracing innovation, and assuming responsibilities. Finally, normative commitment reflects employees’ alignment with general ethical standards in their work, fostering a sense of obligation to remain with the organization. Generativity helps individuals achieve personal fulfillment and a sense of immortality as they approach life’s end (Cordella et al., 2021). Mentoring younger employees is one way to express generativity, which also manifests in leadership, coaching, or participation in organizational social responsibility activities. Within intergenerational interactions, generativity demonstrates older employees’ compassion, responsibility, and tolerance. Employee age and opportunities for creation and development can influence the quality of intergenerational contact, thereby negatively impacting turnover intentions (Climek et al., 2024).

Generativity not only enhances older employees’ organizational commitment but also further strengthens their job dedication. Organizational commitment serves as the psychological bond connecting employees to their organization (Herrera and De Las Heras-Rosas, 2021). Employees with high organizational commitment are more likely to accept organizational norms, identify with organizational goals, and maintain loyalty. Employees with high job dedication exhibit emotional attachment to the organization and consciously adhere to organizational norms, which increases their sense of work focus (Ng, 2023). Emotional commitment is negatively correlated with turnover intention; high organizational commitment leads employees to remain in the current organization due to career utility (Al Balushi et al., 2022). Enhancing organizational commitment increases employees’ job dedication (Brunetto et al., 2013) and elevates job satisfaction (Graf et al., 2016), thereby boosting work vitality.

Generativity, a key psychosocial developmental trait in adulthood, enhances older employees’ organizational commitment and may boost their motivation to work by facilitating knowledge transfer, contribution, influence expression, and responsibility (Wiktorowicz et al., 2022). Organizations can leverage measures to activate older employees’ generativity, monitor and support various expressions of generativity, fulfill employees’ personal growth needs (London and Smither, 1999), establish organizational commitment (Meyer et al., 2002), and thereby enhance older employees’ job dedication.

*H2*: Generativity influences older employees' job dedication by enhancing organizational commitment.

### 2.3 The moderating role of cognitive trust and affective trust

Employees’ proactive attitudes and contributions beyond their job responsibilities constitute a vital source of organizational competitive advantage. In environments marked by heightened uncertainty, institutionalized management struggles to deliver full effectiveness, making trust a critical factor in uniting employee interests, reducing friction, and facilitating organizational operations (Morgan and Zeffane, 2003). A trust-based management orientation fosters sustainable organizational development.

Trust is categorized into cognitive trust and affective trust (McAllister, 1995). Cognitive trust stems from beliefs about another’s reliability and dependability, resulting from rational judgments based on personal attributes, such as competence, integrity, and honesty (Legood et al., 2023). Affective trust, conversely, is rooted in mutual care and concern, reflecting specific emotional bonds between trusting parties and emphasizing the formation of high-quality relationships that transcend economic contracts.

Cognitive trust influences older employees’ generativity and organizational commitment. When supervisors are perceived as trustworthy, older employees view demonstrated generativity as more valuable, thereby increasing their willingness to create knowledge legacies for the organization through intergenerational knowledge transfer. This trust reduces self-protective and defensive behaviors, enabling employees to focus on activities that create value for the organization. Older employees with high cognitive trust may perceive generativity as a valuable behavior, believing it helps demonstrate competence and improve task performance (Wang et al., 2024). Consequently, generativity exerts a stronger positive influence on organizational commitment among older employees with high cognitive trust.

Affective trust is equally important. Based on affective trust, older employees deeply perceive their supervisors’ care and concern, thereby reducing their focus on the costs of generativity behaviors (Wiktorowicz et al., 2022). This trust leads employees to believe supervisors will not negatively evaluate them for underperforming in generativity. For older employees with high affective trust, the perceived costs of generativity are reduced, making them more willing to engage in role-transcending behaviors. Conversely, employees with low affective trust may reduce generativity behaviors due to perceived high costs, thereby undermining organizational commitment.

In summary, both cognitive trust and affective trust amplify the positive impact of generativity on older employees’ organizational commitment. Trust provides psychological safety and a sense of value, making employees more willing to make extra contributions to the organization. Therefore, we propose the following hypothesis:

*H3*: Cognitive trust and affective trust amplify the positive influence of generativity on older employees' organizational commitment. Specifically, for individuals with high levels of both cognitive trust and affective trust, generativity exerts a stronger positive effect on organizational commitment.

**Figure 1:**
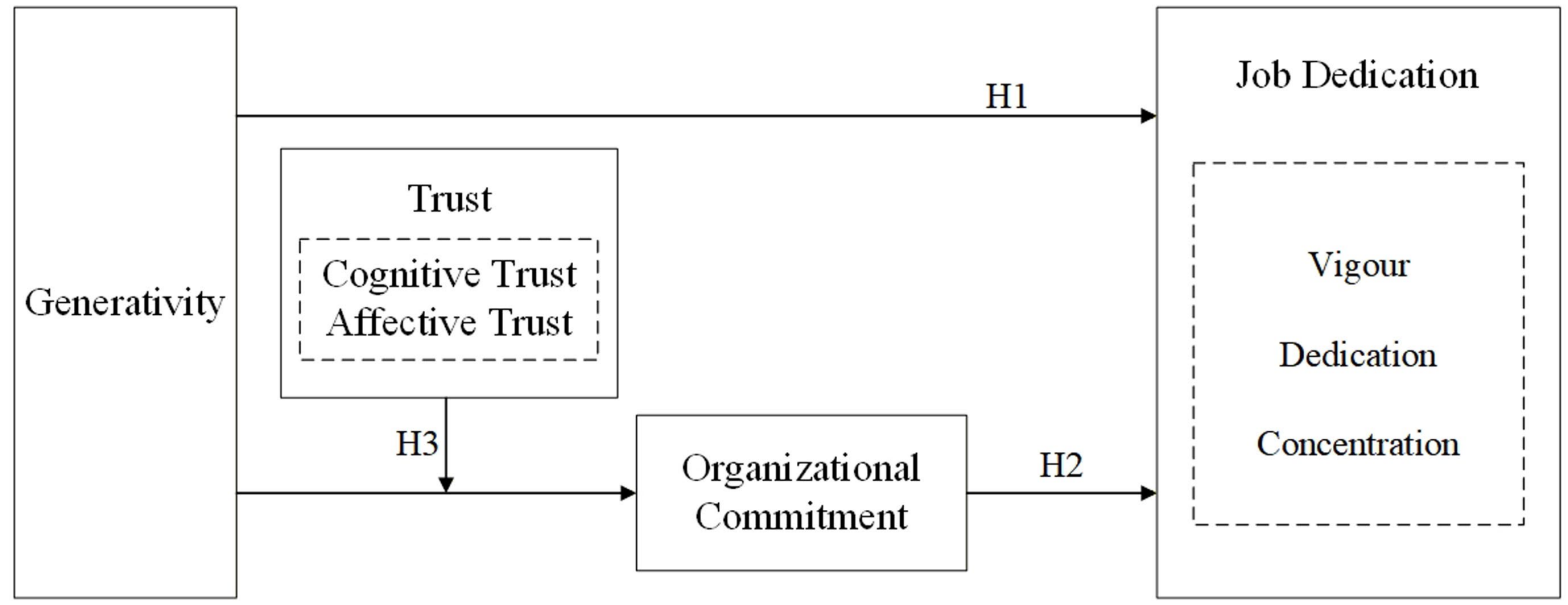
Mechanism of generativity’s influence on older employees’ job dedication: an integrated model.

The conceptual framework depicted in Figure 1 clearly illustrates the relational structure among variables, reflecting the core tenets of the socioemotional selectivity theory: As individuals age, they increasingly prioritize emotional fulfillment and meaning creation. Generativity, as the central mechanism for meaning creation, ultimately enhances job dedication by strengthening organizational commitment. Cognitive trust and affective trust function as boundary conditions within this process.

## 3 Research methods

### 3.1 Research process and sample

This study operationally defines “older employees” as those aged 40 and above who are currently employed. This delineation is based on three considerations: (1) Within the Chinese labor market context, age 40 represents a critical turning point in career development (Athukorala and Wei, 2018); (2) Organizational behavior research commonly uses age 40 as the threshold for classifying older employees (Hirshorn, 1988); (3) Preliminary interviews revealed that employees in this age group commonly begin reassessing career goals and life priorities, aligning with core predictions of the socioemotional selectivity theory. Additionally, to more precisely capture psychological age characteristics, subjective age (participants’ self-perceived age) was measured and included as a control variable in the analysis.

The study employed a multistage stratified sampling method combined with convenience sampling principles. First, we selected four representative provinces from eastern China. Second, we randomly chose three large state-owned enterprises and two private enterprises in each province. Finally, with assistance from human resources departments, we invited eligible employees from the selected enterprises to participate based on predefined age screening criteria. Although the final sample composition was constrained by corporate cooperation willingness, we enhanced representativeness through strict inclusion criteria and multi-regional coverage. Ultimately, 536 questionnaires were distributed to older employees across multiple enterprises and institutions in East China. To ensure data quality, the questionnaire incorporated three types of screening and quality control questions: (1) Attention checks (e.g., “Please select ‘Somewhat disagree’ to confirm you have carefully read this question”), identifying respondents who answered perfunctorily; (2) Logical consistency tests (e.g., asking similar content with different phrasing across sections), detecting internal inconsistencies in responses; (3) Extreme response pattern identification (selecting the same option consecutively over 80% of the time), excluding thoughtless answering patterns. These three quality control mechanisms collectively form a multi-tiered data cleansing process, rather than relying solely on respondents’ self-reported honesty. This quality control process references the multi-method response quality assessment framework (Meade and Craig, 2012), effectively enhancing data reliability and validity. Ultimately, 489 valid questionnaires were obtained, achieving a valid response rate of 91.2%. Detailed employee information is presented in Table 1.

**Table 1:** Demographic status of the participants.

Variables	Categories	Number of participants	Percentage (%)
Gender	Male	247	50.5
	Female	242	49.5
Age	Ages 40–49	254	51.9
	Ages 50–59	209	42.7
	Age 60 and older	26	5.40
Education	Bachelor’s degree	81	16.6
	Master’s degree	334	68.3
	Doctoral degree	74	15.1
Job tenure	10 years or less	21	4.3
	10–15 years	53	10.8
	16–20 years	172	35.2
	21–25 years	168	34.4
	26 years and older	75	15.3

Source(s): Table created by author.

The education levels in the table represent the degree levels of older employees; The table shows the demographic information of the participants in the valid questionnaire.

### 3.2 Measuring tools

This study employed different-point Likert scales to measure distinct constructs, a decision based on the following considerations: (1) Each scale retained the original developers’ scoring formats to ensure cross-study consistency and comparability of measurements; (2) The generativity construct employed a 4-point Likert scale (1 = Strongly Disagree, 4 = Strongly Agree), aligning with the standard scoring method of the McAdams and de St. Aubin scale, which effectively mitigates the midpoint bias tendency among older participants; (3) Organizational commitment, cognitive trust, affective trust, and job dedication all employ 7-point scales (0 = never, 6 = always), consistent with the culturally adapted versions of their original scales, providing finer distinctions. Existing research indicates that when analysis focuses on relationships between variables rather than absolute score comparisons, data from scales with different point numbers can be effectively integrated for analysis (Carifio and Perla, 2008).

Generativity. A 20-item scale developed by McAdams and de St Aubin (1992) was used. The sample question was titled “I try to pass along the knowledge I have gained through my experiences.” The questionnaire was scored on a 4-point Likert scale (1 = not at all, 4 = fully). *α* = 0.778.

Cognitive trust. The Cognitive Affective Trust Scale, developed by Ng and Chua (2006), consisting of eight entries, was used. One of the items, 1–4, was titled Cognitive Trust Scale. The questionnaire was scored on a Likert 7-point scale (0 = never, 6 = always). *α* = 0.77.

Affective trust. The Cognitive Affective Trust Scale, developed by Ng and Chua (2006), consisting of eight items, was used. Among them, 5–8 items were titled Affective Trust Scale. The questionnaire was scored on a Likert 7-point scale (0 = never, 6 = always). *α* = 0.78.

Organizational commitment. The 9-item Organizational Commitment Scale developed by Mowday et al. (1979) was used. The sample question was entitled “I am willing to go above and beyond what is expected to help the unit succeed.” The questionnaire was scored on a 7-point Likert scale (0 = never, 6 = always). *α* = 0.813.

Job dedication. A short version of the 9-item job dedication Scale developed by Schaufeli et al. (2006) was used, consisting of three dimensions of Vigour, Dedication, and Concentration, with three entries per dimension. The questionnaire was scored on a Likert 7-point scale (0 = never, 6 = always). *α* = 0.859.

Control variables. In this study, rank, subjective age, desired retirement age, education, length of service, and health were selected as control variables. Subjective age is measured through seven domains (i.e., family, friends, leisure activities, personality, appearance, mental, and physical) (Kornadt et al., 2018). For each domain and each participant, we calculated a proportional score, where we subtracted a person’s subjective age from a person’s actual age and divided this result by a person’s actual age [(age − subjective age)/age]. We then averaged these scores across the fields to arrive at a final subjective age score.

This study incorporates subjective age (i.e., an individual’s perceived self-age) rather than relying solely on chronological age, primarily based on the core tenets of the socioemotional selectivity theory (SST). According to SST, an individual’s time perspective and motivational priorities are more influenced by perceived remaining time than determined solely by calendar age. For older employees, subjective age better reflects their subjective experience of future time perspective than chronological age. Future time perspective is a key psychological mechanism influencing their work motivation and behavioral choices (Baird et al., 2021). For example, among two 55-year-old employees, one who subjectively perceives themselves as 45 and another who perceives themselves as 65 will, according to SST, exhibit distinct orientations: the former is more likely to adopt a “time-abundant” mindset, pursuing knowledge acquisition and career development; the latter is more likely to adopt a “time-limited” mindset, prioritizing emotional fulfillment and meaning. Within our research framework, incorporating subjective age as a control variable helps distinguish the distinct pathways through which physiological aging and psychological aging influence job dedication, thereby enhancing the model’s explanatory power regarding the heterogeneity among older employees.

### 3.3 Statistical analysis

This study employed a four-stage data analysis process: (1) exploratory factor analysis (EFA) and descriptive statistics were conducted using SPSS 26.0; (2) confirmatory factor analysis (CFA) was performed via Mplus 8.3 to assess the discriminant validity of the measurement model; (3) Pearson correlation analysis and common method bias tests were conducted; (4) *testing moderated mediation effects using* PROCESS Model 7 (5,000 resamples). Specifically, exploratory factor analysis was first conducted on each measurement tool: 17 generativity dimension indicators were grouped into four dimensions; nine organizational commitment indicators into two dimensions; four cognitive trust indicators formed a single dimension; four affective trust indicators formed a single dimension; and nine job dedication indicators were grouped into two dimensions. Next, confirmatory factor analysis using maximum likelihood estimation assessed the model fit and discriminant validity of the measurement models. Subsequently, the means, standard deviations, and correlation coefficients for each variable were calculated, and the common method variance was evaluated using Harman’s one-factor test. Finally, the moderated mediating effect was validated using bootstrap sampling (5,000 repetitions), with 95% confidence intervals reported. This analytical strategy was chosen because it first confirms the structural validity of the measurement tools and then precisely estimates the relationships between variables and indirect effects, ensuring the robustness of the research conclusions.

## 4 Results

### 4.1 Exploratory factor tests for structural validity between variables

The level of structural validity was high. The study was analysed for structural validity using exploratory factor analysis, where 20 entries for generativity were divided into four dimensions, 9 entries for organizational commitment were divided into two dimensions, 4 entries for cognitive trust were one dimension, 4 entries for affective trust were one dimension, and 9 entries for job dedication were divided into two dimensions. The results are shown in Table 2. When validity was tested through factor analysis, the KMO values were all greater than 0.7, with a minimum of 0.759 and a maximum of 0.878, and all of them passed Bartlett’s test (*p* < 0.05). Therefore, it indicates that the structural validity of the variables in this study is high and the sample data are valid for subsequent analyses.

**Table 2:** Exploratory factor analysis results.

Variables	KMO	*X^2^*	*df*	*p*
Generativity	0.837	1385.748	136	0.000
Cognitive trust	0.759	490.984	6	0.000
Affective trust	0.768	539.403	6	0.000
Organizational commitment	0.835	1200.015	36	0.000
Job dedication	0.878	1649.141	36	0.000

Source(s): Table created by author.

### 4.2 Confirmatory factor test of discriminative validity between variables

Good discriminant validity between the main variables. For the validation factor analysis, we used the Shapiro–Wilk test to determine the data’s binary normal distribution. If *p* > 0.05 in the S-W test, then it conforms to a multivariate normal distribution. The data of this study (MVM = 0.0980 *p* = 1.000) were significantly greater than 0.05; therefore, we were able to verify that the data showed a binary normal distribution. As presented in Table 3, the five-factor model fitted well (*X*^2^ = 124.233, *p* < 0.01; CFI = 0.965; TLI = 0.941; RMSEA = 0.058; SRMR = 0.038) (*X*^2^, goodness-of-fit test; CFI, Comparative Fit Index; TLI, Tucker–Lewis index; RMSEA, Root Mean Square Error of Approximation; SRMR, Standardized Root Mean Square Residual) and was significantly different from the other models.

**Table 3:** The results of confirmatory factor analysis.

Models	*X^2^*	*df*	*X^2^/df*	*△X^2^*	RMSEA	SRMR	CFI	TLI
Single-factor model	658.002	58	11.344		0.145	0.146	0.726	0.631
Two-factor model	622.663	57	10.923	35.339	0.142	0.145	0.741	0.646
Three-factor model	587.562	55	10.682	75.101	0.141	0.144	0.757	0.655
Four-factor model	153.074	51	3.001	434.488	0.064	0.041	0.953	0.929
Five-factor model	124.233	47	2.643	28.841	0.058	0.038	0.965	0.941

Source(s): Table created by author.

Synthesize all items into a single potential factor; Consider job dedication as a potential factor and other variables as a potential factor; Take organizational commitment and job dedication, respectively, as potential factors, and other variables, respectively, as potential factors; Combine cognitive trust and affective trust into one potential factor, and take other variables, respectively, as one potential factor; Take each variable as a potential factor. df, degree of freedom.

This study employs a combined approach of procedural and statistical controls to address common method bias (Zhou and Long, 2004). Procedurally, core variable data are collected at three distinct points in time to mitigate the risk of bias associated with single-point measurements. Statistically, the Harman single-factor test is employed to assess common method bias (Podsakoff et al., 2003). The results reveal that adding a method factor to the five-factor model results in a six-factor model (*X*^2^ = 135.901, *p* < 0.01; CFI = 0.958; TLI = 0.928; RMSEA = 0.064; SRMR = 0.055), yet the model fitting index was not significantly enhanced (*△* CFI = −0.007, *△* TLI = 0.13; *△* RMSEA = 0.006, *△* SRMR = 0.017) (Wen-Zhonglin and Hou, 2006). Additionally, the CFA (CFA, Confirmatory Factor Analysis) results demonstrate that the single-factor model is poorly fitted (*X*^2^ = 658.002, *p* < 0.01; CFI = 0.726; TLI = 0.631; RMSEA = 0.145; SRMR = 0.146).

### 4.3 Correlation analysis of each variable

To avoid confounding variables affecting the research findings, this study employed Pearson correlation analysis. The means, standard deviations, and correlation coefficients for each variable are detailed in Table 4. Based on Pearson’s bivariate correlation results, generativity showed significant positive correlations with both organizational commitment and job dedication (*p* < 0.01); generativity also exhibited significant positive correlations with both cognitive trust and affective trust (*p* < 0.01); cognitive trust and affective trust both showed significant positive correlations with organizational commitment and job dedication (*p* < 0.01); and organizational commitment and job dedication showed a significant positive correlation (*p* < 0.01). The corresponding significant *p*-values for all five variables were less than 0.05, indicating significant correlations exist among the five variables studied.

**Table 4:** The mean, standard deviation, and correlation coefficient of each variable.

Variables	1	2	3	4	5	6	7	8	9	10	11
1. Generativity	1	.	.	.	.	.	.	.	.	.	.
2. Cognitive trust	0.425^**^	1	.	.	.	.	.	.	.	.	.
3. Affective trust	0.385^**^	0.635^**^	1	.	.	.	.	.	.	.	.
4. Organizational commitment	0.464^**^	0.526^**^	0.562^**^	1	.	.	.	.	.	.	.
5. Job dedication	0.460^**^	0.388^**^	0.445^**^	0.658^**^	1	.	.	.	.	.	.
6. Level	0.045	0.029	0.013	−0.031	−0.008	1	.	.	.	.	.
7. Subjective age	−0.071	−0.149^**^	−0.100^*^	−0.045	−0.060	−0.189^**^	1	.	.	.	.
8. Expect retirement age	0.067	0.018	0.027	0.067	0.037	−0.410^**^	0.021	1	.	.	.
9. Education degree	0.127^**^	0.042	0.063	0.064	0.162^**^	0.082	−0.187^**^	−0.001	1	.	.
10. Job tennure	0.029	−0.020	0.022	0.110^*^	0.110^*^	−0.134^**^	0.355^**^	0.197^**^	−0.091^*^	1	.
11. Fitness level	0.148^**^	0.093^*^	0.031	0.157^**^	0.094^*^	−0.001	−0.045	−0.064	0.149^**^	−0.123^**^	1
Mean	2.85	4.05	3.77	3.50	3.25	1.49	4.40	55.64	2.03	3.46	3.53
SD	0.41	1.05	1.09	0.92	0.99	0.50	0.69	4.52	0.93	1.02	0.86

Source(s): Table created by author. Note: *p < 0.05, **p < 0.01.

### 4.4 Hypothesis testing

(1) To test Hypothesis 1 (that generativity positively influences job dedication among older employees), we first examined the main effect of generativity. As shown in Table 5(1), controlling for variables, generativity significantly and positively influenced job dedication (*β* = 1.06, *p* < 0.01). This result supports Hypothesis 1.

**Table 5:** The influence of generativity on job dedication.

Predictive variables	Model 1		Model 2		Model 3		Model 4		Model 5	
Constant	0.39		0.34		1.49		2.85		2.84	
	*β*	*t*	*β*	*t*	*β*	*t*	*β*	*t*	*β*	*t*
Generativity	1.06	10.75**	0.99	10.86**	0.46	5.02**	0.61	6.68**	0.58	6.73**
Organizational commitment					0.61	14.88**				
Cognitive trust							0.36	10.20**		
Affective trust									0.38	12.05**
Generativity * cognitive trust							0.15	2.52**		
Generativity * affective trust									0.18	3.01**
Level	−0.09	−1.00	−0.08	−1.01	−0.04	−0.52	−0.06	−0.82	−0.05	−0.75
Subjective age	−0.09	−1.42	−0.08	−1.43	−0.04	−0.75	−0.01	−0.20	−0.02	−0.43
Expect retirement age	−0.01	−0.76	0.00	0.06	−0.01	−0.96	0.00	0.11	0.00	0.20
Education degree	0.11	2.58	0.00	−0.11	0.12	3.19	0.00	0.11	−0.01	−0.40
Job tennure	0.13	3.04	0.11	2.86	0.06	1.71	0.11	2.95	0.09	2.66
Fitness level	0.03	0.60	0.11	2.57	−0.04	−1.01	0.10	2.43	0.12	3.13
*R* ^2^	0.24		0.24		0.48		0.38		0.42	
*△R* ^2^	0.24		0.24		0.24		0.01		0.01	
*F*	21.55		21.71		55.17		6.37		9.09	

Source(s): Table created by author. Note: *p < 0.05, **p < 0.01.

(2) To test Hypothesis 2 (that generativity influences job dedication through organizational commitment), we employed Bootstrap sampling (5,000 repetitions) to examine the mediating effect. As shown in Tables 5(2) and (3), generativity significantly and positively influenced organizational commitment (*β* = 0.99, *p* < 0.001); simultaneously, organizational commitment significantly influenced job dedication (*β* = 0.61, *p* < 0.001). Mediational analysis (Table 6) revealed that the indirect effect of generativity on job dedication via organizational commitment was 0.60 (95% CI [0.45, 0.77]), with the interval not containing zero, indicating a significant mediating effect. These results supported Hypothesis 2 (Table 7).

**Table 6:** The test results of the mediating effect.

Mediating path	Effects	Effect value	Standard deviation	95% confidence interval
Generativity → organizational commitment → job dedication	Total effect	1.06	0.10	[0.87 1.26]
	Direct effect	0.46	0.09	[0.28 0.64]
	Indirect effect	0.60	0.08	[0.45 0.77]

Source(s): Table created by author.

**Table 7:** The results of moderated mediating effect tests.

Mediating path	Moderating variables	Effect value	Standard deviation	95% confidence interval
Generativity → organizational commitment → job dedication	Low cognitive trust	0.27	0.08	[0.10 0.43]
	High cognitive trust	0.47	0.09	[0.31 0.66]
	Low affective trust	0.24	0.08	[0.07 0.40]
	High affective trust	0.47	0.09	[0.31 0.67]

Source(s): Table created by author.

(3) To test Hypothesis 3 (that cognitive trust and affective trust moderate the effect of generativity on organizational commitment), we examined the moderating effects of each trust type separately. As shown in Table 5(4), the interaction term between generativity and cognitive trust significantly influenced organizational commitment (*β* = 0.15, *p* < 0.01); Table 5(5) indicates that the interaction term between generativity and affective trust was also significant (*β* = 0.18, *p* < 0.01). According to the moderated mediation process (Edwards and Lambert, 2007), the table reveals that when the mean of cognitive trust increases or decreases by one standard deviation, the difference in the indirect effect of generativity on job dedication reaches 0.2 (*p* < 0.01), indicating a significant difference. When the mean of affective trust increases or decreases by one standard deviation, the difference in the indirect effect of generativity on job dedication reaches 0.23 (*p* < 0.01), also showing a significant difference. These findings support Hypothesis 3.

## 5 Discussion

To cope with the ageing of the workplace, fully exploit older human resources, and improve the job dedication of older employees, this study explored the effects of generativity on older employees and its underlying mechanisms. It was found that generativity positively affects job dedication by increasing organizational commitment. Furthermore, high levels of cognitive and affective trust strengthen the relationship between generativity and organizational commitment, which in turn enhances job dedication. This study is an expansion of previous research on generativity and job dedication. Most previous researchers have proposed a positive effect of generativity on the job dedication of older employees based on social exchange theory (Qaiser and Abid, 2022); however, some researchers have also proposed a weakening effect of generativity on job dedication. Theoretically, the researchers proposed that to clarify the relationship between generativity and job dedication, attention needs to be paid to the differences in the needs and preferences of older employees due to their age, and that such differences can lead to different outcomes (Van Buren III et al., 2011). The outcome effects of generativity are subject to a variety of situations and conditions. One is that the particular differences in the needs preferences of older employees are ignored (Löckenhoff and Carstensen, 2004); two, generativity is not equally effective in any culture or for any organisation (Wiktorowicz et al., 2022); and three, there are strict boundary conditions for both the positive and negative impacts of generativity (Thomas and Tee, 2022). Thus, the relationship between generativity and job dedication among older employees will vary from particular employees and from culture to culture, and its effects may have strict boundary conditions. Grounded in socioemotional selectivity theory, this study directly focused on older employees and examined the mediating role of organizational commitment in the relationship between generativity and job dedication. It also validated the moderating roles of cognitive trust and affective trust.

### 5.1 Theoretical contributions

First, the empirical study of this study deepened the understanding of the impact of generativity. Several empirical studies and reviews on generativity mention that generativity’s impact effects need to focus on the heterogeneous needs of older employees due to ageing (Van Buren III et al., 2011). Related studies have questioned the underlying assumption of a positive effect of generativity (based on the social exchange theory that giving without a reward reduces giving) (Cropanzano and Mitchell, 2005) and fail to explain managerial practices where generativity is still implemented without a reward (Randall, 2020). Older employees have a reduced external demand for late career, and long-term goals are unattractive to older employees. More attractive for older employees is to play the role of a mentor, make a short-term impact, and achieve short-term goal achievement (Apostu et al., 2022). This study puts the relationship between generativity and job dedication in the context of age-specific needs and confirms the positive effect of generativity on the job dedication of older employees based on socioemotional selectivity theory with full consideration of future time, short-term goals, and limited resources, which enriches the understanding of the impact of generativity.

Second, this study reveals the intrinsic mechanism of the effect of generativity on job dedication among older employees, confirming the mediating role of organizational commitment, and forming a useful addition to the relevant research on the warmth effect. Much of the previous research on the warmth effect has emphasised the positive emotional experiences that result from the intrinsic drivers of altruistic behavior; for example, research in the field of psychology has shown that altruistic behavior is motivated by the sense of satisfaction and wellbeing that the behavior brings (Weinstein and Ryan, 2010). This study demonstrates that generativity enables older employees to gain lasting influence and provides them with opportunities to contribute to younger colleagues’ development. Through this process, older employees develop stronger organizational commitment as they find greater meaning and purpose in their work roles. Our empirical findings confirm that organizational commitment serves as a key psychological mechanism through which generativity translates into increased job dedication among older employees.

Third, the study explores the boundary conditions under which generativity positively affects organizational commitment, empirically testing the moderating role of cognitive trust and affective trust. Our findings show that when older employees have high levels of cognitive trust in their organization and leaders, the positive relationship between generativity and organizational commitment is strengthened. Similarly, high levels of affective trust amplify this relationship. This finding addresses previous calls to identify contextual factors that determine when and for whom generativity leads to positive outcomes (Thomas and Tee, 2022). Our results provide empirical evidence that trust serves as a critical contextual factor that enhances the positive impact of generativity on older employees’ organizational commitment and subsequent job dedication.

### 5.2 Practical contributions

This article finds that generativity improves the job dedication of older employees through organizational commitment and that cognitive trust and affective trust can enhance this positive effect, which enlightens organizational managers.

First, organizations should create structured opportunities for older employees to engage in generative activities that align with their desire for meaningful short-term contributions. These activities should be designed to provide immediate feedback and recognition to satisfy older employees’ socioemotional needs. Organizations can facilitate knowledge-sharing sessions, experience-based problem-solving forums, and formalized wisdom transfer programs that allow older employees to contribute their expertise while receiving visible appreciation for their contributions.

Second, organizations should strengthen the organizational commitment of older employees by recognizing and valuing their generative contributions. This can be achieved through formal recognition programs, creating visible pathways for their continued influence within the organization, and ensuring their generative efforts are meaningfully integrated into organizational processes. In collectivist contexts, particularly, emphasizing how their contributions benefit the collective and are recognized by the group can significantly enhance commitment.

Finally, organizations should actively develop both cognitive and affective trust between older employees and their leaders. For cognitive trust, this involves demonstrating competence, consistency, and transparency in decision-making processes. For affective trust, leaders should show genuine care for older employees’ wellbeing, acknowledge their career histories, and demonstrate respect for their accumulated wisdom. Our findings suggest that when both forms of trust are high, the positive impact of generativity on organizational commitment is maximized.

### 5.3 Research gaps and future prospects

First, although our theoretical framework provides evidence for the temporal ordering of the variables, the cross-sectional study does not exclude the possibility that job dedication may positively influence the formation of generativity. For example, when older employees have high levels of job dedication, they may allow themselves to be more accountable and have more status and influence in their organisations, and an increase in generativity may occur. Therefore, to demonstrate a causal relationship between generativity and job dedication, future research needs a longitudinal study design to demonstrate this.

Second, the managers in this study were located at the top level of the unit of employment, and the effects and pathways of trust in different levels of managers on the outcome variables may be different. Further research could expand the management hierarchy to include senior, middle, and junior managers, and explore the differences in the effects and mechanisms of employees’ cognitive and affective trust in managers at different levels on the psychology and behavior of older employees. For example, in terms of the mechanism of influence, employees’ affective trust in middle and senior managers affects employees’ affective commitment to the organisation, which in turn affects performance. In contrast, affective trust in junior unit of employment managers may affect the direct relationship between the two parties, e.g., through LMX, which affects the employees’ direct performance.

Third, in terms of the generalizability of the findings, the role of generativity varies considerably by industry, as the data in this study were obtained from units of employment, and the specific functions of generativity were not disaggregated in the analysis. In the case of financial firms, the focus might be on role models and career guidance, with a particular emphasis on risk control (Yang and Li, 2018). Future research could collect cross-industry data for analysis to enhance the generalizability of the findings.

Fourth, this study focuses on the role of generativity in influencing job dedication among older employees in a unit of employment. However, in order to more fully understand this complex relationship, we believe it is necessary to integrate current research on the relationship between older employees’ generativity and dedication through a meta-analysis and to explore the role of additional moderating and mediating variables in the relationship between older employees’ generativity and dedication. This meta-analysis will help quantify effect sizes and reveal consistency and heterogeneity across studies.

Finally, due to the limited capacity of personal collection, this study selected older employees from several enterprises as well as institutions and related departments in East China as study participants; therefore, the results of this article can only represent the older employees in eastern China, and the conclusions of the study may not be universally applicable in China as well as in other geographic scopes. Future research could expand the scope of the survey and improve the coverage of the sample so that the results of the impact of generativity on the job dedication of older employees could be more generalizable.

## 6 Conclusion

This study, grounded in the socioemotional selectivity theory, focuses on older employees in eastern China. It examines the “warmth effect” of generativity on job dedication, along with the mechanisms and boundary conditions of this relationship, offering a new interpretive perspective on their previously unclear connection. Findings reveal that generativity indirectly influences job dedication through organizational commitment, while cognitive trust and affective trust amplify generativity’s positive impact on older employees’ organizational commitment.

Theoretically, this study overcomes limitations of social exchange theory by constructing an integrated model of generativity’s influence on job dedication from an age-specific needs perspective. It reveals the critical mediating role of organizational commitment and validates the differential moderating effects of cognitive versus affective trust within a collectivist cultural context, thereby expanding the application boundaries of socioemotional selectivity theory in organizational behavior. Practical contributions include providing organizations with actionable trust-building strategies. Managers can leverage structured platforms to unlock older employees’ generativity potential, transforming “idle experience” into “wisdom inheritance” and maximizing the value of aging human resources. Limitations include a sample restricted to enterprises in eastern China, insufficient examination of industry-specific moderation effects, and the inability of the three-wave longitudinal design—though superior to cross-sectional studies—to fully establish causal relationships. Future research should expand cross-cultural comparisons, deepen industry-specific analyses, and employ longer-term tracking designs to examine dynamic evolutionary relationships among variables.

## Data Availability

The original contributions presented in the study are included in the article/Supplementary material, further inquiries can be directed to the corresponding author.
