# Correction: The impact of older employees' generativity on job dedication: a socioemotional selectivity perspective

**DOI:** 10.3389/fpsyg.2026.1793660

**Published:** 2026-02-09

**Authors:** Zhenxing Gong, Yongqi He, Miaomiao Li

**Affiliations:** 1 School of Business, Liaocheng University, Liaocheng, China; 2 Business School, Beijing Information Science and Technology University, Beijing, China

**Keywords:** generativity, job dedication, organizational commitment, socioemotional selectivity theory, trust

Author [Miaomiao Li] was erroneously assigned to affiliation [School of Economics and Management, Beijing Information Science and Technology University, Beijing, China]. This affiliation has now been removed from the published article.

Author [Zhenxing Gong] was erroneously assigned to affiliation [Department of Communication Arts, University of Wisconsin-Madison, Madison, WI, United States]. This affiliation has now been removed from the published article.

Affiliation [Business School, Beijing Information Science and Technology University, Beijing, China] has now been added to author [Miaomiao Li].

The original version of this article has been updated.

