# Supplementary material for: The impact of older employees’ generativity on job dedication: a socioemotional selectivity perspective
Source: Front Psychol. 2026 Jan 20;17:1703410. doi: 10.3389/fpsyg.2026.1703410 (PMC12864090; doi:10.3389/fpsyg.2026.1703410)
Supplement: Supplementary file 1 [file Table_1.DOCX]

Supplementary Material

**Supplementary Figure 1.** Mechanism of generativity's influence on older employees' job dedication: An integrated model

**Appendix A.** Generativity Scale Items (McAdams & de St Aubin, 1992)

1. I try to pass along the knowledge I have gained through my experiences.

2. I do not feel that other people need me.

3. I think I would like the work of a teacher.

4. I feel as though I have made a difference to many people.

5. I do not volunteer to work for a charity.

6. I have made and created things that have had an impact on other people.

7. I try to be creative in most things that I do.

8. I think that I will be remembered for a long time after I die.

9. I believe that society cannot be responsible for providing food and shelter for all homeless people.

10. Others would say that I have made unique contributions to society.

11. If I were unable to have children of my own, I would like to adopt children.

12. I have important skills that I try to teach others.

13. I feel that I have done nothing that will survive after I die.

14. In general, my actions do not have a positive effect on others.

15. I feel as though I have done nothing of worth to contribute to others.

16. I have made many commitments to many different kinds of people, groups, and activities in my life.

17. Other people say that I am a very productive person.

18. I have a responsibility to improve the neighborhood in which I live.

19. People come to me for advice.

20. I feel as though my contributions will exist after I die.
